# Induced Hydrophilicity and Oxidation of β‑Iron Disilicide Films by Varying Ar Plasma Etching Durations

**DOI:** 10.1021/acsomega.6c07509

**Published:** 2026-08-20

**Authors:** Nattakorn Borwornpornmetee, Thanatcha Kardkaew, Rattanaporn Sansit, Chonnarong Kaewsai, Kriettisak Srisom, Rungrueang Phatthanakun, Pattanaphong Janphuang, Hideki Nakajima, Boonchoat Paosawatyanyong, Tsuyoshi Yoshitake, Nathaporn Promros

**Affiliations:** † Department of Physics, King Mongkut’s Institute of Technology Ladkrabang (KMITL), Bangkok 10520, Thailand; ‡ School of Physics, Institute of Science, Suranaree University of Technology (SUT), Nakhon Ratchasima 30000, Thailand; § Synchrotron Light Research Institute (SLRI), Nakhon Ratchasima 30000, Thailand; ∥ Kamnoetvidya Science Academy (KVIS), Rayong 21210, Thailand; ⊥ Department of Advanced Energy Science and Engineering, 12923 Kyushu University, Fukuoka 816-8580, Japan

## Abstract

Induced hydrophilicity and oxidation of β-iron disilicide thin films using 300 W microwave Ar plasma for 5–15 min were investigated experimentally and statistically. Plasma diagnostics revealed cold plasma characteristics with a stable electron temperature (0.287–0.283 eV) and a gradually accumulated electron density over time. X-ray photoelectron spectroscopy analysis revealed reduced adventitious carbon and an increased presence of hydrophilic oxide and hydroxyl groups forming on vacant bonds accumulated over time on the activated surface after sputter ion bombardment. The film morphology demonstrated a roughness of 4.10 nm after 15 min of etching from the original roughness of 2.52 nm due to the shape evolution of nanoscale spikes and valleys on the surface after ion impact. Owing to these tandem surface morphology changes and chemical modifications, the contact angle decreased from 96.13° (hydrophobic) to 69.08° (hydrophilic) after 15 min, while maintaining the mechanical properties intact. This active surface with a contact angle under 70° has implications for further fabrication of polymer interlayers and other metallic coatings onto the top of the surface as a possible use case in the vehicular and electronic industries.

## Graphical abstract

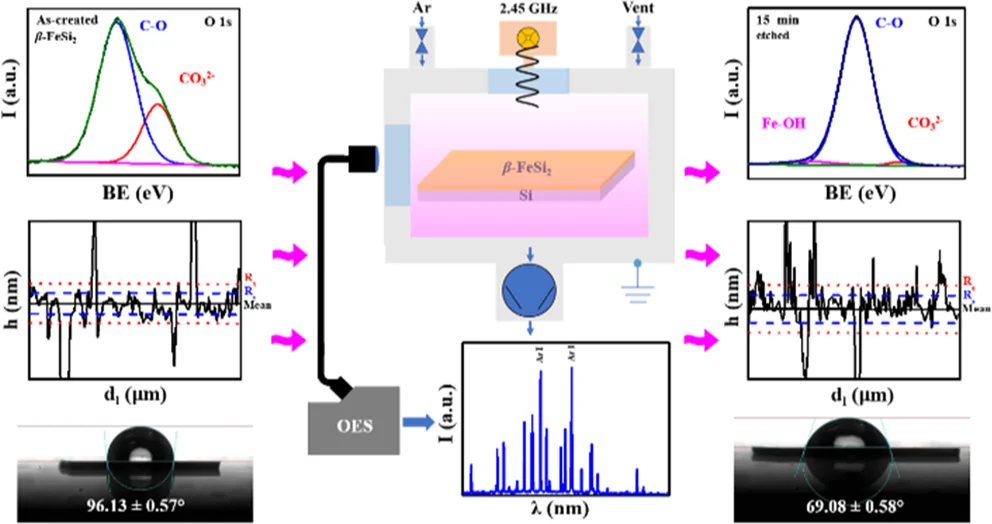

## Introduction

Self-cleaning surface technology can be developed using superhydrophilic and superhydrophobic surfaces. The superhydrophobic self-cleaning surface is inspired by the natural phenomenon, where water droplets roll off the lotus leaf’s surface, whose contact angle exceeds 150° due to a combination of hydrophobic chemical components and nanoscale roughness on the surface, according to the Cassie–Baxter model.^1–4^ Meanwhile, a self-cleaning surface can also be inspired by a pitcher plant with a micro-grooved surface structure and a tissue surface coated with hydrophilic nectar.^5,6^ The increased roughness of the hydrophilic surface decreases its contact angle, as designated by the Wenzel model, making the pitcher plant surface superhydrophilic.^5,6^ As a result, water spreads out with a contact angle near zero degrees and slides down the pitcher plant sac.^5,6^ When the surface of the material can create the same phenomena, it can be applied for self-cleaning since the contaminants become attached to the water and move away together.^5,6^ To create innovations and develop products with self-cleaning surfaces, new materials that are environmentally friendly and naturally abundant with superhydrophobic or superhydrophilic properties are crucial.^7^ Previous studies and reports have revealed that orthorhombic β-iron disilicide (β-FeSi_2_) is an outstanding composite for various applications, such as optical diodes, hydrophobic surfaces, and hard coatings.^8–11^ The direct optical bandgap of β-FeSi_2_ is 0.85 eV, and the indirect bandgap is 0.78 eV, correlating with the wavelengths used in optical fiber-based telecommunications (1.3 and 1.5 μm).^8–11^ The characteristics of β-FeSi_2_ also include an absorption coefficient of over 10^5^ cm^–1^ under a 1.5 eV photon energy.^8–11^ Moreover, β-FeSi_2_ is an Fe–Si-based composite composed of materials that are both nontoxic and abundant on Earth and possess an even surface.^8–11^ In addition to its usual advantages of being toxic-free and abundant in nature, which FeSi_2_ shares with TiO_2_ and SiO_2_ films, FeSi_2_ also shows hardness nearly that of dimond-like carbon (DLC) while also possessing a high modulus, which is tougher than that of the other common self-cleaning materials.^8^ Additionally, FeSi_2_ should present better substrate adhesion when coated on an alloy or steel substrate due to its Fe composite nature.^12,13^ The nearly neutral wetting state of the Fe–Si composite also gives it the potential to be modified to be either hydrophilic or hydrophobic, depending on the technique used for its modification.^8^ These characteristics imply the potential application of β-FeSi_2_ as a hard coating material with a superhydrophobic surface. In our previous work, β-FeSi_2_ thin films were created on a silicon substrate through epitaxial growth at 600 °C substrate heating, which is 200 °C lower than the usual substrate heating used for β-FeSi_2_ coating.^14^ The influence of conventional thermal annealing on the β-FeSi_2_ film’s surface created through facing-target sputtering (FTS) was studied, revealing that the hydrophobic surface of unannealed β-FeSi_2_ turned hydrophilic after annealing at 600 °C.^15^ Afterward, the researchers investigated the wettability of β-FeSi_2_ surfaces epitaxially created onto a silicon substrate using radio frequency (RF) sputtering onto the substrate at different heating temperatures of 500, 560, and 600 °C and found that all the films created post-contact angle had a hydrophobic range of 99.25°, 99.80°, and 102.00°.^16^ The researchers also studied the influence of substrate heating on the wetting characteristics of β-FeSi_2_ formed through FTS.^8^ The created β-FeSi_2_ surfaces showed hydrophobic behavior with a contact angle of 91.15° for a substrate temperature of 525 °C, increasing to 103.80° when a substrate temperature of 660 °C was used.^8^ Notably, surface modification methods play a crucial role in the modification of superhydrophobic surfaces, which may possess nanoroughness, low surface energy, and air pockets. However, studies on the wettability of β-FeSi_2_ thin films and their potential as superhydrophobic coatings are still lacking, including those on the surface modification of β-FeSi_2_ films through plasma etching, which has barely been studied.

There are various plasma sources for plasma apparatuses, including RF, DC, microwave, plasma jet, and dielectric barrier discharge configurations.^17–19^ Low-frequency plasma sources are often the least effective for etching, as no selective surface etching can be performed. Plasma is categorized into two types according to the source: a source that operates in a vacuum using a low- and a high-pressure source that operates at atmospheric pressure.^17–19^ Microwave plasma is generally more efficient than RF plasma in terms of high-density radical generation efficiency and yield per watt of the active substrate.^17–19^ Therefore, the plasma-induced chemical reaction rate is high.^17–19^ Due to the absence of a high-voltage sheath with an ion sputtering wall and electrode inside, the microwave variant has lower material contamination in the chamber than the RF variant.^17–19^ A typical microwave reactor is based on the conduction of energy into a large multimode cavity (chamber) through a waveguide and quartz window.^17–19^ The plasma density was improved while also giving more control through a finer etch rate.^17–19^ Plasma etching can modify the surface characteristics of a material by causing physical roughening and/or chemical interaction between plasma ions and the surface. This process reduces contamination and improves the surface qualities.^17–21^ CF_4_, O_2_, Ar, and N_2_ gases are the most popular medium gases for plasma etching at present.^22–32^ After reviewing, CF_4_ gas is a commercial nontoxic etchant, which is mainly used for Si-based materials and has the ability to elevate the hydrophobicity of the material’s surface.^22–24^ The CF_4_ gas can induce fluorination of the surface, which increases the surface energy and, hence, the contact angle.^22–24^ O_2_ can also contribute to the modification of the surface through oxidation–reduction reactions.^25–27^ O_2_ plasma has the potential to modify the surface roughness, including enhancing the hardness, nanoscratch resistance, and wear properties after O_2_ etching.^25^ Research has shown that O_2_ plasma etching can alter the wetting state of certain materials from hydrophilic to hydrophobic and vice versa.^26,27^ Meanwhile, N_2_ plasma can enhance the integrity of the surface structure and mechanical properties through bonding by nitriding.^28–30^ Hence, N_2_ plasma is suitable for metallic film modification due to its advantages, such as being more economical, less destructive, and less contaminating.^28–30^ N_2_ plasma also possesses the unique characteristics of a nitriding effect, which enhances the durability and electrical properties of metallic surfaces.^28–30^ In inert gas plasma, such as Ar, ions interact with the material by colliding and breaking the bonds of impurities, such as hydrocarbons, and evaporating them with the energy received from the ions hitting the surface without relying on chemical reactions.^31–33^ In addition, the effect of plasma etching also causes surface activation by the functional groups remaining on the material’s surface after high-energy ion collisions.^20,21^ With an ion energy lower than the binding energy of oxide bonding, the ions would merely impart their energy to the surface or partially break the bond, leading to an active surface that could bond and form new oxide or oxygen-based radicals easily.^34^ If ions possess energy surpassing that of oxide bonding, the Ar plasma can completely remove oxide through the collision of ions with enough energy to volatilize the bond.^34^ Reactive species can also induce oxidation and the formation of numerous oxides that may contain hydroxyl radicals.^34^ According to the literature related to plasma surface modification, the time taken to etch the sample with plasma is an important factor for controlling the hydrophilicity of the material. Ru et al. reported the effect of varying the etching time of Ar plasma on the properties of polyvinyl chloride (PVC) sheets.^31^ The findings reveal that PVC has hydrophobic wetting properties and a contact angle of more than 100°, which tends to decrease with increasing etching time, resulting in a decrease in hydrophilic groups such as carbon and chlorine, while the oxides become more hydrophilic, resulting in an increase in the surface energy until the water contact angle is less than 25° after 8 min of etching. Zajíčková studied the etching of a polycarbonate surface with Ar gas plasma and found that a longer etching time can promote the surface energy and hydrophilicity from a hydrophilic surface with a contact angle of 70° to a superhydrophilic surface with a contact angle below 10°.^32^ Moreover, Ting et al. studied the etching of an aluminum surface using Ar gas plasma at different etching times.^33^ They found that the plasma at 1000 W etching for 30 s could reduce the high hydrophilic contact angle from ∼20° to ∼13°, and when etched for a longer time of 10 min, the contact angle of 13° was reduced to approximately 4°, resulting in a superhydrophilic surface. Using the above-mentioned approach, this study determined that changing the duration of Ar microwave plasma etching can reduce the contact angle to the superhydrophilic level.

Accordingly, Ar plasma etching could be the key to enhancing the hydrophilicity of β-FeSi_2_ surfaces formed by sputtering. The researchers aim to optimize the etching parameters of Ar plasma to adjust the contact angle of water on β-FeSi_2_ surfaces for self-cleaning purposes by altering the etching time. This work reports a change in the plasma parameters used to generate microwave Ar plasma through the plasma diagnosis of glow discharge emission spectra and a change in the chemical composition of β-FeSi_2_ films, along with the mechanism behind such a change. The mechanical properties are also reported alongside the wetting properties to demonstrate the film’s surface integrity for coating applications. Longer plasma etching times are expected to make the surface of β-FeSi_2_ films more hydrophilic, resulting in different Ar gas plasma parameters breaking more chemical bonds on the surface due to physical collisions with higher energy ions. These factors affect the composition by reducing the amount of contaminated carbon and producing more hydrophilic oxide and hydroxyl radical groups through induced oxidation, as well as increasing the roughness, which could affect the wetting model of the surface. For all these reasons, these changes are likely to result in an alteration in the film’s contact angle from the original. Identification of the appropriate conditions for developing new products consisting of easy-to-find, abundant in nature, and environmentally friendly materials for use in several possible fields, like self-cleaning coating, friction-reducing coating for automotive parts, advanced electronics engineering, dental implant adhesion layer, and water vapor barrier coating for food packaging. To the best of our knowledge, no studies or reports currently exist on the effect of Ar plasma etching time on the wettability and oxidation behavior of β-FeSi_2_ films.

## Experimental Section

### Preparation of the Si Substrate

A Si(111) wafer (10–20 Ω·cm resistivity and 0.260 mm thickness) was selected as the substrate for the preparation of β-FeSi_2_ films. High-purity (4N) FeSi_2_ targets (Toshima Manufacturing Limited Company) were mounted inside the target holder for the deposition of each β-FeSi_2_ film. First, the Si(111) wafer underwent ultrasonic cleaning (Branson 2510, Yamato Scientific Co., Ltd.) with acetone, methanol (2N purity, FUJIFILM Wako Pure Chemical Corp.), and deionized water. Each cleaning step lasted 5 min in an ultrasonic cleaner, ensuring the removal of organic residues and solvents. Hydrofluoric acid (HF) (1% diluted in deionized water, Morita Chemical Industries Co., Ltd.) was then used to eliminate surface oxide. After oxide removal, any excess HF was removed with deionized water, and the wafer was thoroughly dried using high-purity nitrogen gas (6N purity, Iwatani Corporation).

### Sputtering Process for β-FeSi_2_ Films

The cleaned Si substrate was placed in a holder 75 mm apart from a pair of FeSi_2_ targets inside the direct-current sputtering apparatus in a clean room. The gases inside the sputtering chamber were vacuumed to a baseline pressure of 10^–5^ Pa using a rotary vacuum pump (Alcatel Japan, 2030C series) alongside a turbomolecular pump (Osaka Vacuum, TG1300 series). Ar gas (4N high purity, Iwatani Industrial Gases Corp.) was then introduced under a gas feeding rate of 15 mL/min, controlled by a mass flow meter (Kojima Instruments Incorporated), achieving a working pressure level of 1.33 × 10^–1^ Pa for deposition. During the entire deposition process, the heating temperature of the stage behind the substrate was maintained at 600 °C using a heater equipped with a digital temperature controller (OMRON Corp., E5CN series). A direct-current voltage of 1 kV supplied by a Micro Denshi HD1K-30N power source, with a current stabilized at 1.5 mA, was applied to the sputtering target. The entire deposition process was performed over 24 h.

### Etching of β-FeSi_2_ Films with Ar Plasma

The β-FeSi_2_ film surfaces were etched using a microwave Ar plasma. Plasma etching was conducted in a microwave plasma system that included a low-vacuum plasma cleaning chamber (Diener Electronic GmbH & Co. KG, Pico), a magnetron microwave generating module (Diener Electronic GmbH & Co. KG, MWG 1200 series), and a gas system connected to the cleaner. The β-FeSi_2_ films were cut into smaller pieces prior to etching, then cleaned using a methanol solution in the sample preparation room, and moved onto the station inside the working chamber of the plasma system. A low-vacuum environment was achieved by using a vacuum pumping apparatus (ULVAC Technologies Inc., DIS-251 series) to evacuate the chamber until the pressure was reduced to < 2 Pa. Ar gas was then delivered to the inside of the low-vacuum chamber under a fixed gas flow rate of 50 sccm to maintain the working pressure inside the chamber at 50 Pa. The inner temperature inside the chamber was stabilized at 20 °C. Each β-FeSi_2_ sample was etched for 5, 10, and 15 min under a constant plasma power of 300 W. An optical emission spectrometer (OES) was simultaneously attached to the chamber to analyze the plasma parameters in the etching chamber during the etching process. The emission spectra from Ar atoms, collected using a spectrometer (Ocean Optics, HR4000) connected via an optical fiber cable with a 400 nm numerical aperture, were processed using OceanView 2.0 software (Ocean Optics). This method provided insights into the effects of plasma parameters on Ar plasma characteristics, which directly influence particle energy during etching, since the surface modifications during etching can damage surface crystallinity, chemical reactions, or changes in material properties.^35,36^ To achieve optimal etching results, plasma parameters like gas pressure, generating power, and etching time must be tailored to the specific applications. Key plasma characteristics, including the temperature of electrons (*T*_e_) and density of electrons (*n*_e_), can be evaluated using OES spectroscopic methods.^35,36^

### Measuring Methods

This study employed X-ray photoelectron spectroscopy (XPS), X-ray diffractometry (XRD), cold field-emission variant of scanning electron microscopy (FE-SEM), noncontact atomic force microscopy (AFM), contact angle measurements, and nanoindentation as the analytical methods to investigate the properties of unetched and plasma-etched β-FeSi_2_ films. The crystal structure, including orientation, was analyzed using XRD (Rigaku, SmartLab series) under a conventional 2θ–θ mode within a scanning range of 20° to 70°. The scanning was done in continuous mode with a scanning speed of 5°/min, moving at 0.02° per step. The optical alignment comprised a rotating anode X-ray source (model: PhotonMax high-flux 9 kW; Cu Kα radiation with a wavelength of 1.5406 Å), incident slit box of 1.000 mm, length-limiting slit of 10 mm, detector (model: D/teX Ultra 250), receiving slit box #1 of 1.000 mm, and receiving slit box #2 of 1.125 mm. XPS (Kratos Analytical, Axis Ultra DLD series) was utilized to determine the chemical composition of both the unetched and Ar plasma-etched films. The atomic concentrations were processed and quantified using CasaXPS software (Casa Software Ltd.). The X-ray source was Al Kα running at an energy level of 1486.6 eV under 30 W of power (20 mA and 15 kV). During measurements, the background pressure was 8 × 10^–7^ Torr and the base pressure was 9 × 10^–9^ Torr. The analysis area of high-resolution spectra was set at ≈ 300 × 700 μm^2^. The takeoff angle of electron emission and the incidence angle in relation to the film’s surface were set at ≈ 90 and 0°, respectively. The pass energies were 160 and 40 eV for the wide and narrow scans, respectively. No Ar^+^ ions were used during the measurements, and charge compensation was employed to supply electrons using a neutralizer. The charge neutralizer used for all analyses with charge neutralization (0.4578 A) via the electron flood gun (and optional ion gun) system integrated into the Kratos instrument was monitored using the C 1s signal. The work function was evaluated by measuring the low-energy electron cutoff spectra at BL3.2U in the Synchrotron Light Research Institute, Thailand. The vacuum ultraviolet light (monochromatized He I, 21.22 eV) was used to excite the photoelectrons, with the spectra measured using a Scienta R4000 analyzer on the sample negatively biased at room temperature. The binding energy was referenced from the Fermi edge of gold, with a total energy resolution of about 200 meV based on the broadening of the Fermi edge. The base pressure was 4 × 10^–10^ mbar inside the analysis chamber. The work function (WF) was used as a reference to correct the binding energy of the XPS spectra, with the C–C peak in the C 1s spectra adjusted to the eV energy (289.58 – WF), as presented in Table 1.

**Table 1:** WF and Corrected C 1s Peak for the Sputtered *β*-FeSi_2_ Films Etched by Ar Plasma at 300 W for Different Durations of 5, 10, and 15 min

sample name	cutoff	PE	WF	corrected C 1s C–C	measured C 1s C–C	BE shift
gold (for calibration)	17.0698	21.22	4.1502	-	-	-
β-FeSi_2_ as-deposited	18.2009	21.22	3.0191	286.56	286.35	0.21
Ar-etched at 5 min	17.2508	21.22	3.9692	285.61	284.97	0.64
Ar-etched at 10 min	17.1691	21.22	4.0509	285.53	284.99	0.54
Ar-etched at 15 min	17.1854	21.22	4.0346	285.55	285.04	0.51

The properly shifted XPS spectra were fitted using a Gaussian/Lorentzian function with a ratio of 70:30. The 2p spectra used a Shirley background, while the 1s spectra used a linear background. Fe 2p spectra were multiplet plots with full-width at half-maximum (FWHM) within the range of 1 to 1.5 eV. The relative sensitivity factor and peak label were based on Kratos library and Handbook of X-ray photoelectron spectroscopy.^37^ The surface morphologies and the cross-sectional features of all β-FeSi_2_ thin films were detected using an FE-SEM (Hitachi, SU8230 series). Surface images were captured at 100,000 times magnification, and cross-sectional images were recorded at 50,000 times magnification, both at a 5 kV accelerating voltage and a 10 μA current. Pressure in the chamber was maintained at 2 × 10^–4^ Torr, and the working distance was 2.1 mm. The electron beam is produced using a cold field-emission gun. The root-mean-square roughness (*R*_RMS_) on the film’s surface of β-FeSi_2_ films was evaluated using an AFM (Park System, XE-120 series) operating in the noncontact mode. Scanning was performed over an area of 5 × 5 μm^2^. The scanning rate was 0.35 Hz in two directions. The *R*_RMS_ values were derived from the topographical data using Gwyddion software (General Public License). The wetting characteristics of the β-FeSi_2_ surfaces were investigated using a contact angle meter (Dataphysics, OCA20 Series), with deionized (DI) water as the probe liquid to evaluate the wetting properties. During measurements, the DI water droplet was dispensed at a rate of 0.5 μL/s until the droplet reached a volume of 5 μL. The sessile drop was taken in static mode at the immediate moment of contact between the droplet and the film. The image of the contact angle between the DI water and the surface of the films was fitted using SCA 20 Software, based on the image processing of the captured image. The mechanical features of the unetched and etched β-FeSi_2_ film surfaces were investigated using a nanoindentation machine equipped with a Berkovich indenter (Bruker Hysitron, Ti Primier) to acquire a plot representing the change in penetration depth with respect to the applied indentation load. Using an applied load of 3 mN, the maximum penetration depth of the probe onto the β-FeSi_2_ films was limited to a 10% indentation proportion of the individual thickness of each film (roughly 55 nm) to suppress the substrate effect.^38,39^ The dwelling time of the measuring process was 12 s for each testing point of 5 points per sample. The unloading portions of the load-depth curves were used to calculate the average nano-hardness values (*H*_nano_) and average reduced modulus values (*E*_r_) from five discrete areas on all β-FeSi_2_ films.^38,39^

## Results and Discussion

### Plasma’s Analytical Discussion

A study on the optical emission spectrum of low-vacuum Ar gas plasma generated through the microwave frequency used in surface etching with a plasma power of 300 W at different etching times of 5, 10, and 15 min revealed spectral lines. These included excited Ar atoms at wavelengths of 693.98, 704.61, 725.26, 736.28, 747.95, 761.15, 770.13, 792.44, 799.23, 809.35, 824.49, 849.49, 864.87, and 909.62 nm, which also had oxygen atoms excited at wavelengths 775.00, 839.78, and 919.87 nm, as well as hydrogen atoms at a wavelength of 653.76 nm, as shown in Figure 1. The spectral intensity ratio of Ar peak wavelengths at 761.15 and 809.35 nm from the databank of the National Institute of Standards and Technology was selected to calculate *T*_e_ using the two-emission-spectra ratio method.^20,40^ The calculation revealed that the etching using Ar gas plasma produced by a microwave frequency of 300 W for 5 min had a *T*_e_ value of 0.287 eV, which remained mostly constant at around 0.286 and 0.283 eV at 10 and 15 min etching times, respectively. The Ar gas plasma etching for 5, 10, and 15 min resulted in *n*_e_ values of 3.973 × 10^17^, 3.977 × 10^17^, and 4.027 × 10^17^ cm^–3^ using Stark broadening, indicating properties consistent with cold plasma at low vacuum.^20^ The slight decrease in *T*_e_ but an increase in *n*_e_ over time was due to more particles transferring energy during collisions to stimulate ionization through particle momentum adjustment in a manner similar to our previous work, but under different circumstances. This is because the trend was caused by plasma saturation over an increasingly longer duration, rather than adding energy, since the power was fixed.^20^ This relative stability is due to the extremely high frequency of microwaves, which causes the energy distribution of plasma to be almost time independent.^41^

**Figure 1:**
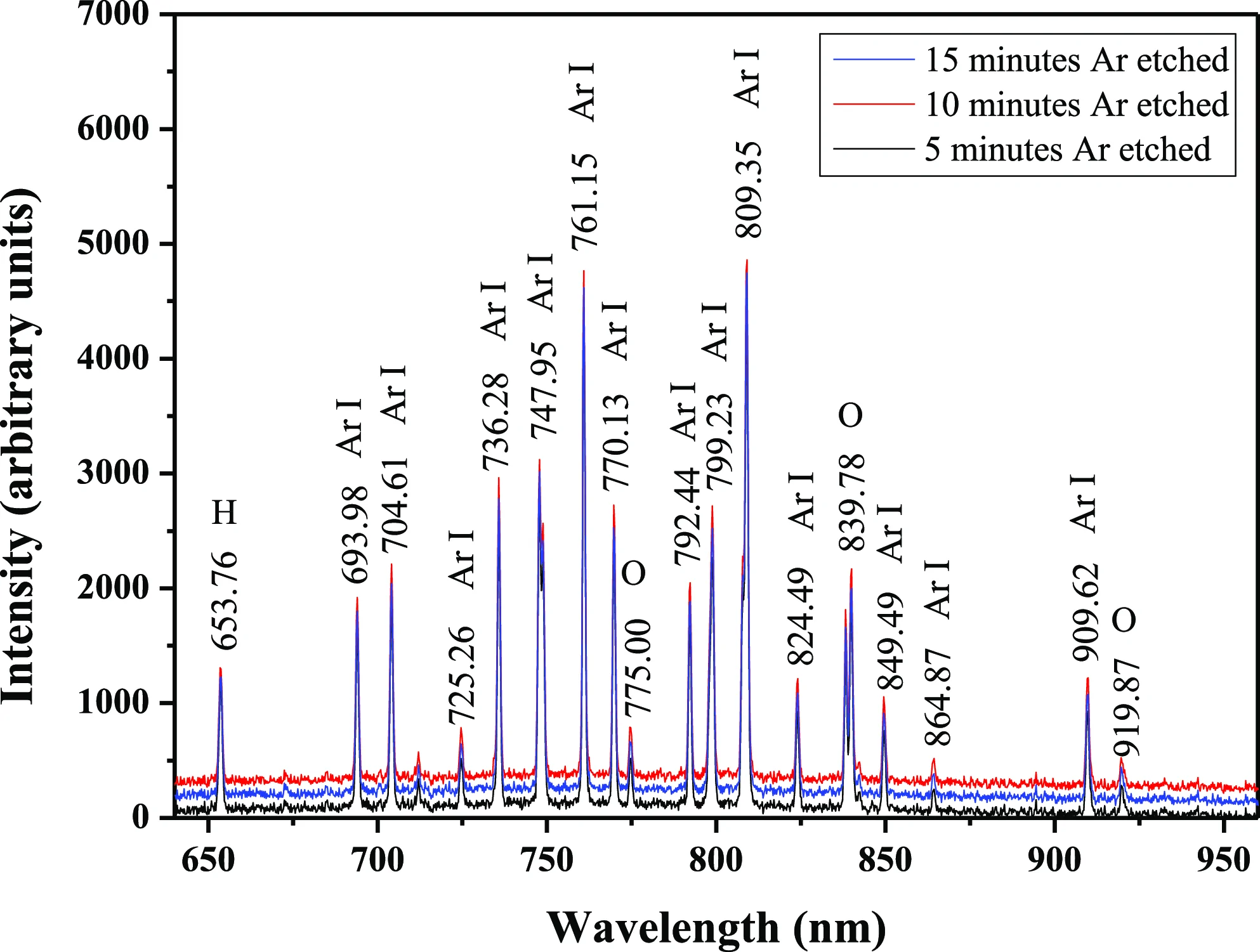
Optical emission spectra of microwave-based Ar plasma at 300 W for different durations of 5, 10, and 15 min.

### Analytical Discussion of Chemical Composition

The wide-scan spectra from the XPS measurements of the unetched β-FeSi_2_ thin films and films etched at different times are displayed in Figure 2, while the narrow-scan spectra of Fe 2p, Si 2p, C 1s, and O 1s are shown in Figures 3, 4, 5, and 6, respectively. The wide-scan XPS spectra identified peaks corresponding to Fe 2p and Si 2p along with C 1s and O 1s, which were used to calculate the atomic concentrations in percentage terms using a proportion of the corrected peak area for a specific element for the summation of the corrected peak area for all elements (corrected area = raw area/relative sensitivity factor).^42,43^ The calculated results of the wide scan of the β-FeSi_2_ film surface are presented in Table 2, which indicate an inverse relationship between the percentage of O 1s and C 1s in each β-FeSi_2_ film. With an increase in the etching duration, the percentage value of C 1s decreased while that of O 1s increased. XPS analysis revealed that the surface composition of all β-FeSi_2_ thin films also contained a large amount of carbon and oxygen in addition to Fe and Si. These elements are common contaminants, especially in materials containing metals, as exposure to environmental air promotes contamination.^44,45^ β-FeSi_2_ is vulnerable to oxidation, which facilitates the development of an oxide layer on the β-FeSi_2_ film’s surface.^45,46^ The derived fitting parameters from the narrow-scan spectra of Fe 2p, Si 2p, C 1s, and O 1s are presented in Tables 3, 4, 5, and 6, respectively. The C 1s peak in Figure 5 and Table 5, especially C–C (may overlap with C–Si), is caused by the spontaneous carbon layer, which could also be easily formed on materials exposed to the atmosphere, while inorganic carbonate can be a byproduct of the oxide group when exposed to CO_2_.^44,45^ These spontaneous carbon layers are often associated with hydrophobic properties. Under low-vacuum microwave plasma etching with high-energy Ar ions, these ions react with the surface of the films, removing organic carbon by volatilizing the carbon atoms.^20^ Oxide groups are also recognized as one of the main factors contributing to the hydrophilicity of the etched surface. Figure 6 and Table 6 display the component peaks of the O 1s peak, representing the hydroxyl and oxide groups. The C–O peak and the inorganic carbonate (overlapping with the oxide group) peak were observed when unetched. The inorganic carbonate peak was reduced significantly when first exposed to 5 min of plasma etching, likely due to the selective etching of the oxide group and its byproduct that could superpose with its peak. Then, the peak slightly increased with a longer etching time.^47^ The C 1s spectrum also showed the change, where the broad peak of inorganic carbonate (likely a combination of both silicon carbonate and iron carbonate) became narrower after etching, implying a similar occurrence. Meanwhile, the surface metal hydroxyl (likely overlapped with adsorbed H_2_O/O_2_ and defected state due to unnatural large FWHM) peak becomes noticeable with an etching duration of 10 min, in contrast to prior conditions where the peak might overlap with the large organic oxide peak. However, the hydroxyl peak slightly decreased for films etched for 15 min compared to those etched for 10 min due to the cessation of oxide/hydroxyl formation from overexposure, causing the ion bombardment to also remove the dangling hydroxyl radical and oxide layer.^47^ Based on the loss of the peak area of Si–O in relation to the peak area of other Si 2p variants depicted in Si 2p (see Figure 4 and Table 4) spectra, which only slightly reduced as etching proceeded, it is clear that Si–O removal is more prevalent than the others. These additional hydrophilic oxide groups and hydroxyl radicals implied by SiO_2_ (and potentially overlapping hydroxyl groups) may originate from trace amounts of oxygen and air in the Ar gas and low-vacuum environment in the microwave plasma chamber, along with the reactive groups remaining on the plasma-etched surface.^20^ The surface reacts with oxygen and hydrogen from the environment or impurities in the plasma, leading to the formation of an oxide group.^20^ The microwave variant of plasma tends to possess a lower ion energy as a trade-off for higher plasma density, as implied by the OES results. Hence, the etching process of the microwave plasma likely relied on cumulative interactions.^17–19^ When the initial ion impact did not contain enough energy to break the bond, the ions would only transfer their energies to the surface, which gave the plasma impurity a chance to form new oxides and radicals.^34^ Meanwhile, the C–O and Fe–OH bonds became less prominent after 15 min of etching due to the longer process of mildly breaking the native oxide bond on cumulative ion collision that left more vacant active species.

**Figure 2:**
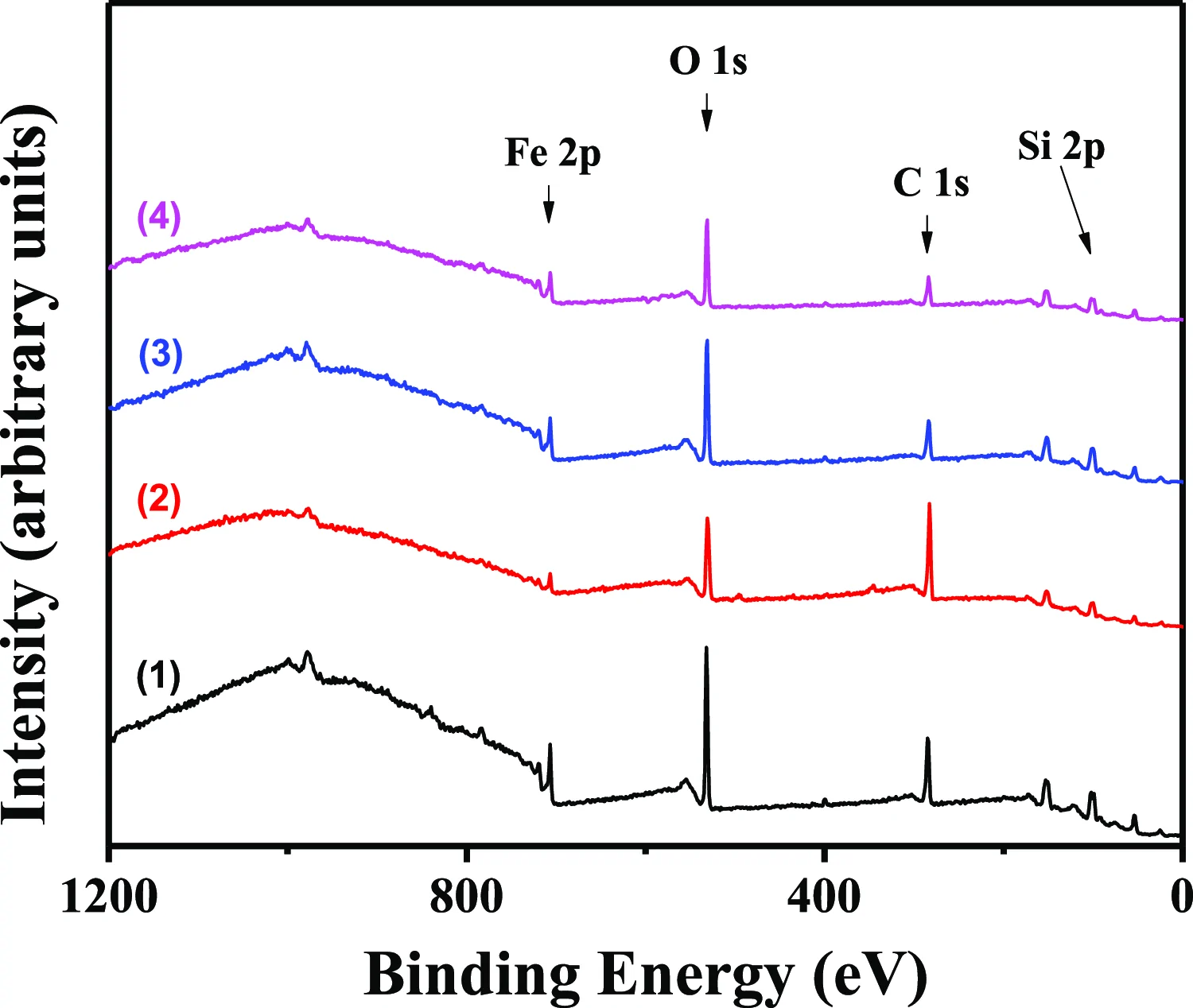
Wide spectra of β-FeSi_2_ films under various conditions: (1) as-coated β-FeSi_2_ films, (2) after 5 min of etching, (3) after 10 min of etching, and (4) after 15 min of etching.

**Figure 3:**
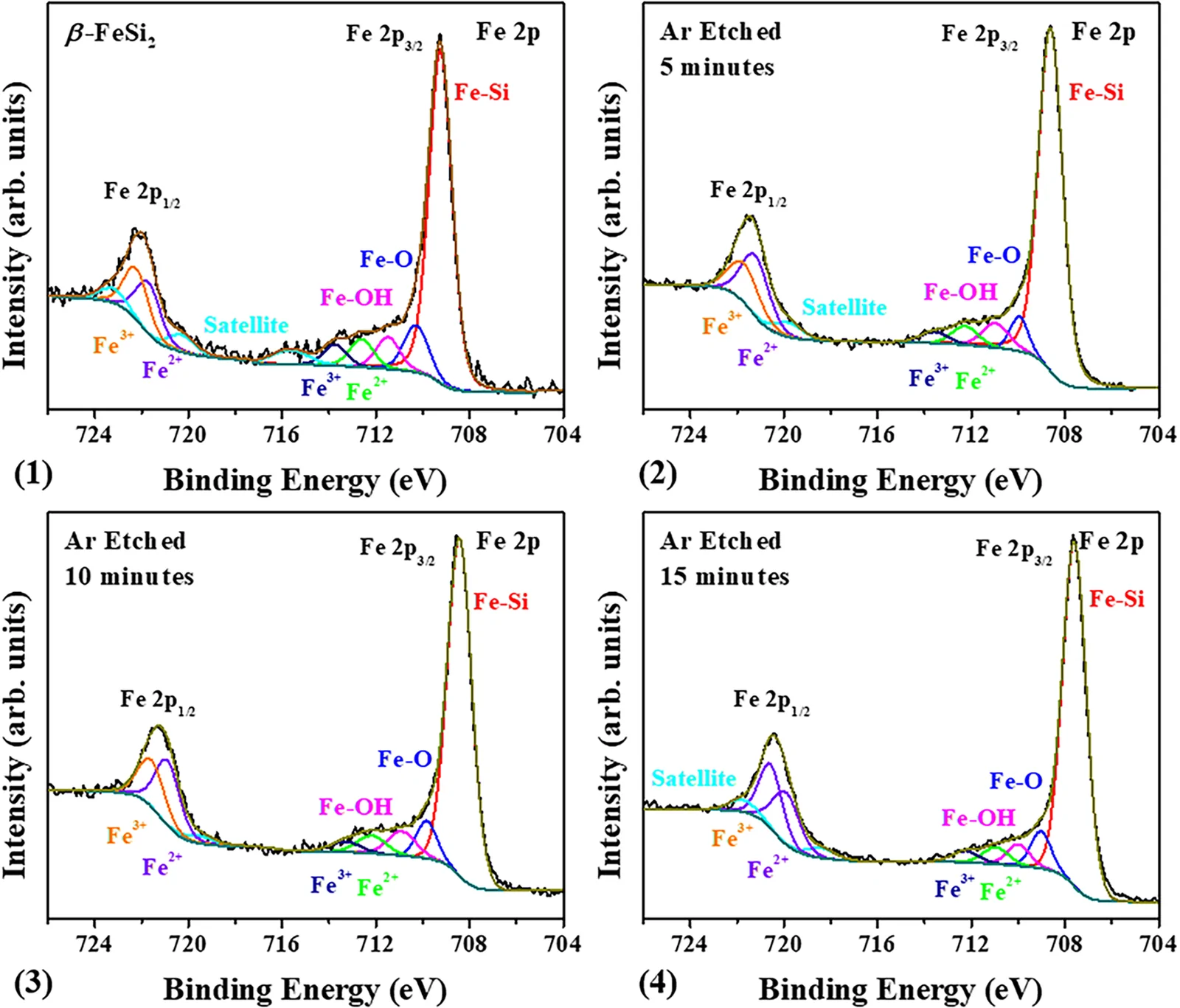
XPS spectra in narrow scan for Fe 2p of the (1) unetched β-FeSi_2_ films compared to the films after Ar plasma etching for durations of (2) 5, (3) 10, and (4) 15 min.

**Figure 4:**
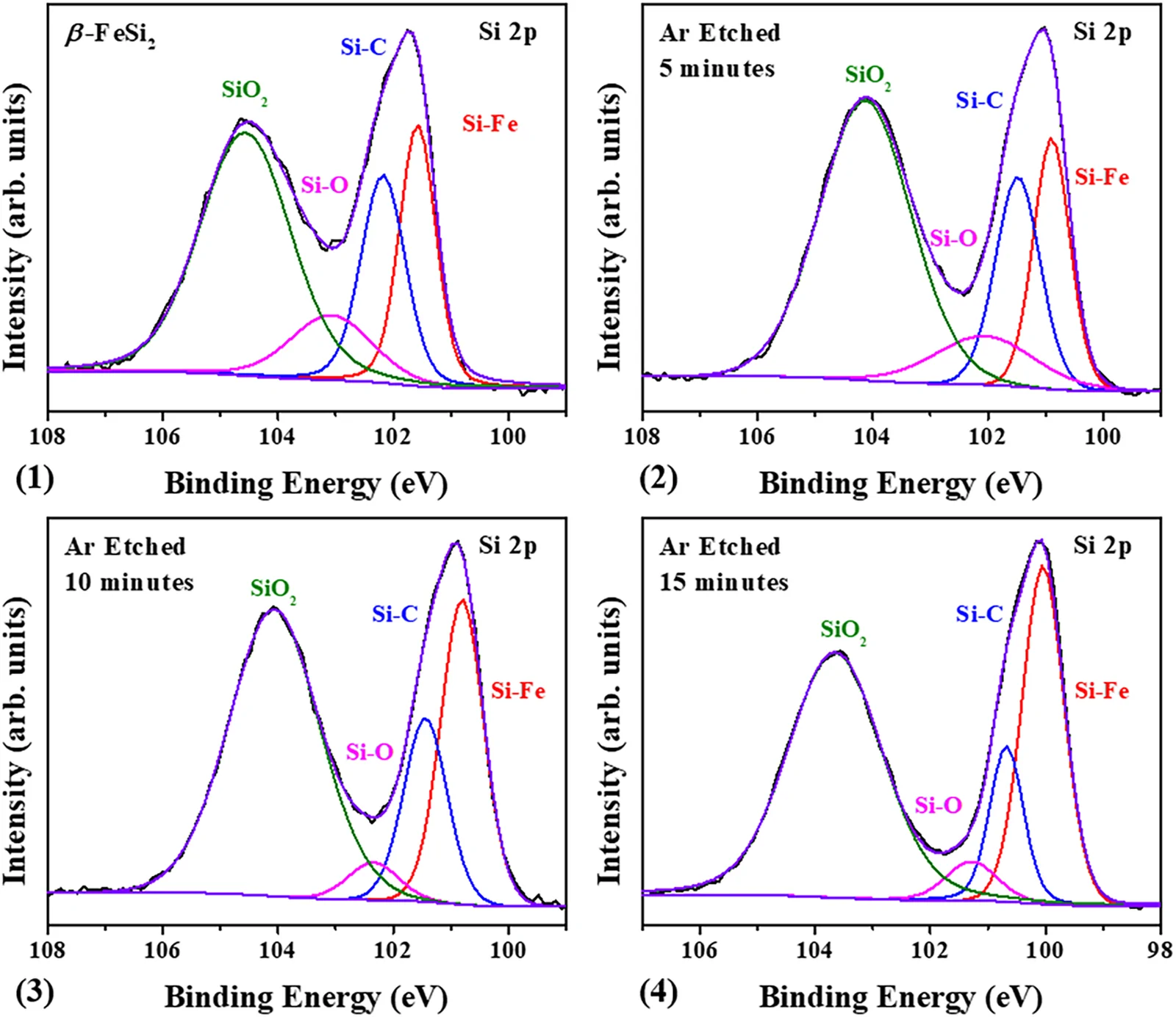
XPS spectra in narrow scan for Si 2p of the (1) unetched β-FeSi_2_ films compared to the films after Ar plasma etching for durations of (2) 5, (3) 10, and (4) 15 min.

**Figure 5:**
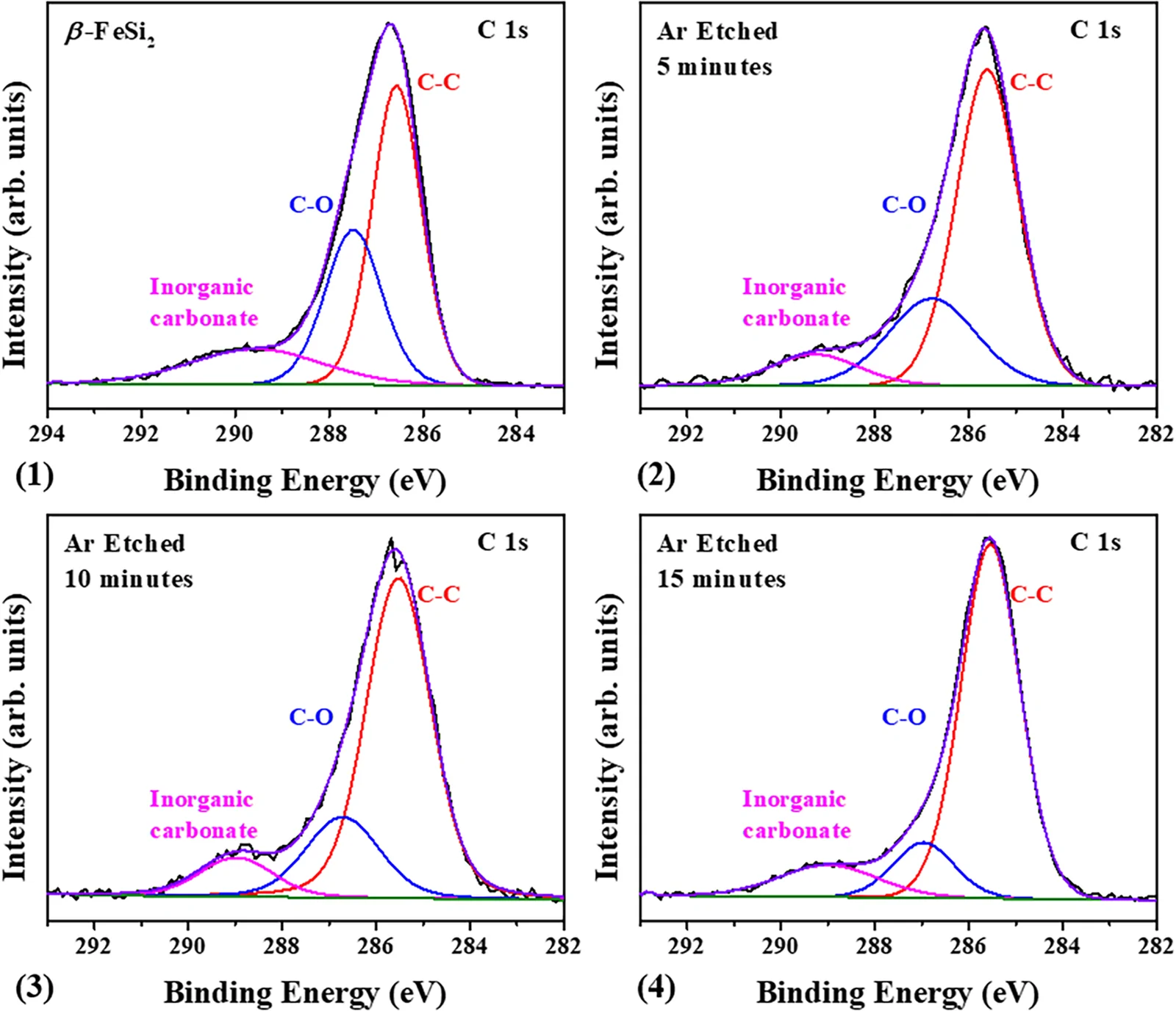
XPS spectra in narrow scan for C 1s of the (1) unetched β-FeSi_2_ films compared to the films after Ar plasma etching for durations of (2) 5, (3) 10, and (4) 15 min.

**Figure 6:**
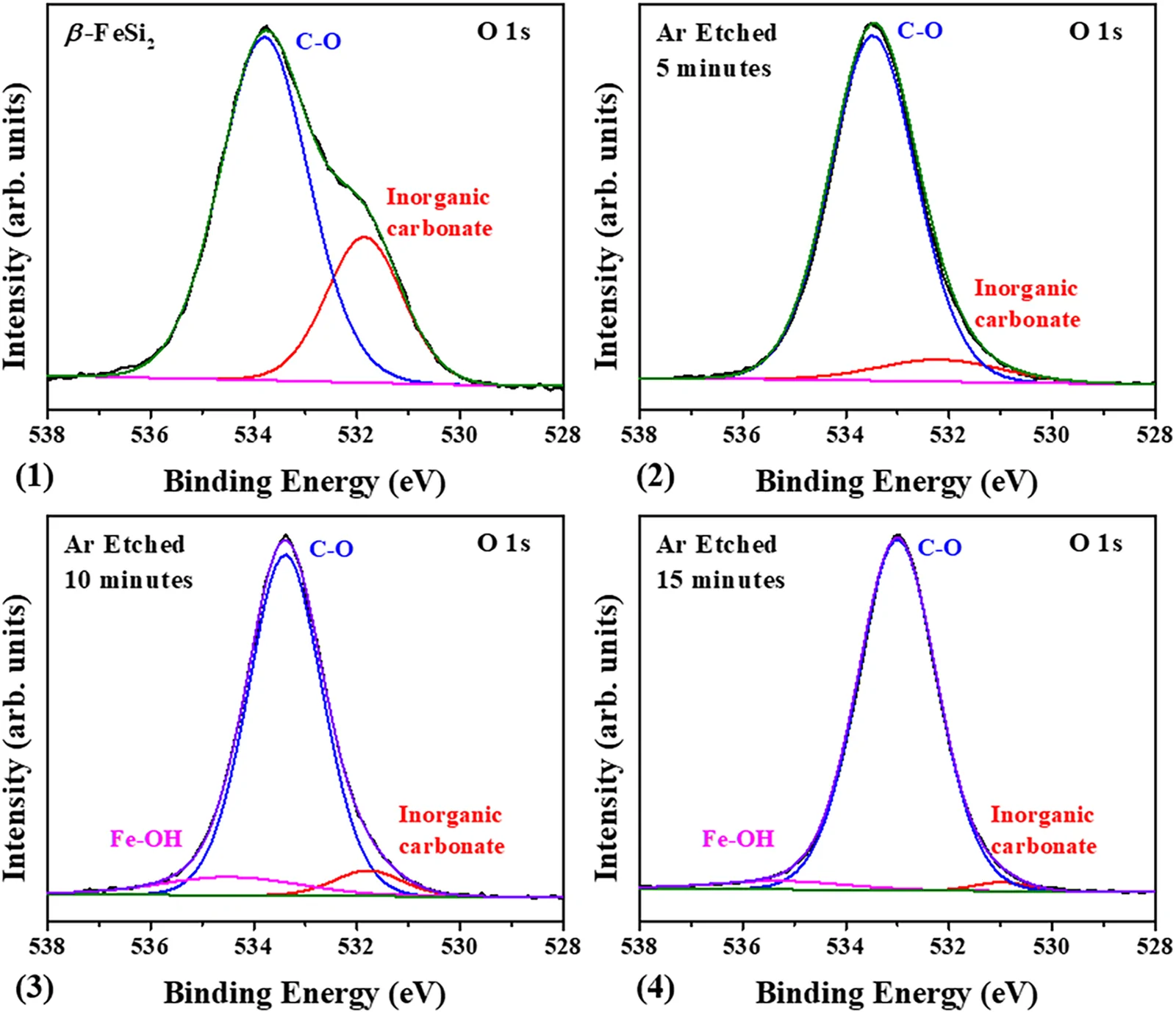
XPS spectra in narrow scan for O 1s of the (1) unetched β-FeSi_2_ films compared to the films after Ar plasma etching for durations of (2) 5, (3) 10, and (4) 15 min.

**Table 2:** Results Derived from the Wide XPS Spectra Representing the Chemical Composition of *β*-FeSi_2_ Surfaces under Various Conditions

	at%			
sample	Fe	Si	C	O
unetched	5.40	14.05	58.70	21.85
5 min	15.37	24.19	34.33	26.10
10 min	16.51	25.73	27.99	29.78
15 min	18.22	24.32	28.27	29.19

**Table 3:** Results Derived from the Narrow XPS Spectra of Fe 2p Representing the Chemical Bonds and their Positions + FWHMs for *β*-FeSi_2_ Films under Each Etching Condition

	ASD		5 min		10 min		15 min	
bond	position	FWHM	position	FWHM	position	FWHM	position	FWHM
Fe–Si	709.03	1.17	707.98	1.21	707.89	1.20	707.10	1.18
Fe–O	710.07	1.23	709.32	0.89	709.29	1.00	708.52	1.00
Fe–OH	711.27	1.20	710.33	1.36	710.36	1.28	709.48	1.13
Fe^2+^ (Fe 2p3/2)	712.40	1.21	711.62	1.30	711.64	1.32	710.46	1.28
Fe^3+^ (Fe 2p3/2)	713.53	1.17	712.93	1.38	712.75	1.41	711.75	1.35
satellite	715.45	1.79						
satellite	720.07	1.32	719.15	1.41	719.02	1.36	717.89	1.39
Fe^2+^ (Fe 2p1/2)	721.50	1.22	720.57	1.36	720.35	1.32	719.41	1.33
Fe^3+^ (Fe 2p1/2)	722.01	1.22	721.05	1.53	721.05	1.33	720.06	1.15
satellite	723.05	1.32					721.26	1.40

**Table 4:** Results Derived from the Narrow XPS Spectra of Si 2p Representing the Chemical Bonds and Their Positions + FWHMs for *β*-FeSi_2_ Films under Each Etching Condition

	ASD		5 min		10 min		15 min	
bond	position	FWHM	position	FWHM	position	FWHM	position	FWHM
Si–Fe	101.37	0.74	100.25	0.75	100.26	0.88	99.54	0.86
Si–C	101.96	0.91	100.84	0.91	100.9	0.89	100.17	0.69
Si–O	102.87	1.68	101.42	2.04	101.81	1.02	100.78	1.05
SiO_2_	104.36	1.86	103.49	1.88	103.53	1.85	103.15	1.92

**Table 5:** Results Derived from the Narrow XPS Spectra of C 1s Representing the Chemical Bonds and Their Positions + FWHMs for *β*-FeSi_2_ Films under Each Etching Condition

	ASD		5 min		10 min		15 min	
bond	position	FWHM	position	FWHM	position	FWHM	position	FWHM
C–C	286.35	1.24	284.97	1.56	284.99	1.62	285.04	1.48
C–O	287.28	1.42	286.14	2.20	286.17	1.85	286.46	1.53
inorganic carbonate	289.34	3.31	288.64	2.20	288.44	1.84	288.5	2.49

**Table 6:** Results Derived from the Narrow XPS Spectra of O 1s Representing the Chemical Bonds and Their Positions + FWHMs for *β*-FeSi_2_ Films under Each Etching Condition

	ASD		5 min		10 min		15 min	
bond	position	FWHM	position	FWHM	position	FWHM	position	FWHM
inorganic carbonate	531.64	1.74	531.54	2.83	531.24	1.69	530.48	1.22
C–O	533.59	2.02	532.83	1.96	532.85	1.55	532.49	1.79
Fe–OH					533.92	3.20	534.76	3.02

### Analytical Discussion on the Structure

According to the XRD analysis of β-FeSi_2_ thin films and those etched under the same power at 300 W in Figure 7, the etching time was different at 5, 10, and 15 min for deviations in the crystal structure when etching the surface with Ar gas plasma generated from the microwave measurements of a 2θ–θ scan from 20° to 70°. Rietveld analysis was performed on the XRD patterns of β-FeSi_2_ using the GSASII software, and the parameters are shown in Table 7. Figure 7(1) and 7(2) show that both XRD patterns demonstrate peaks of β­(202/220) at about 29.1° and Si(111) at about 28.5°, including a small peak of β(021) at 26.2° and β­(404/440) at 60.3°, which are consistent with the characteristics of β-FeSi_2_ reported in previous research, as well as the phase data from mp-149 for Si and mp-1714 for orthorhombic FeSi_2_.^48–50^ The results in Table 8 also show that the films were under compressive strain and yield typical lattice parameters, albeit slightly larger.^51^ The spectra after etching with Ar gas plasma for different durations do not exhibit any significant changes. Since the XRD measurement relies on detecting the X-ray beam that diffracts from the structure of the sample, the presence of a defect layer could slightly deter the X-ray beam trajectory.^52^ Therefore, the removal of the surface defect and contaminant through plasma etching endows the sample with a slightly better X-ray beam propagation due to the reduction of surface-related diffraction. In contrast, structural degradation caused by a long etching duration may create more defects. Hence, the slight fluctuation may be due to the change in defects on the surface, as the etching rarely causes a shift in the bulk structure, as detected by the 2θ–θ mode.^52^

**Figure 7:**
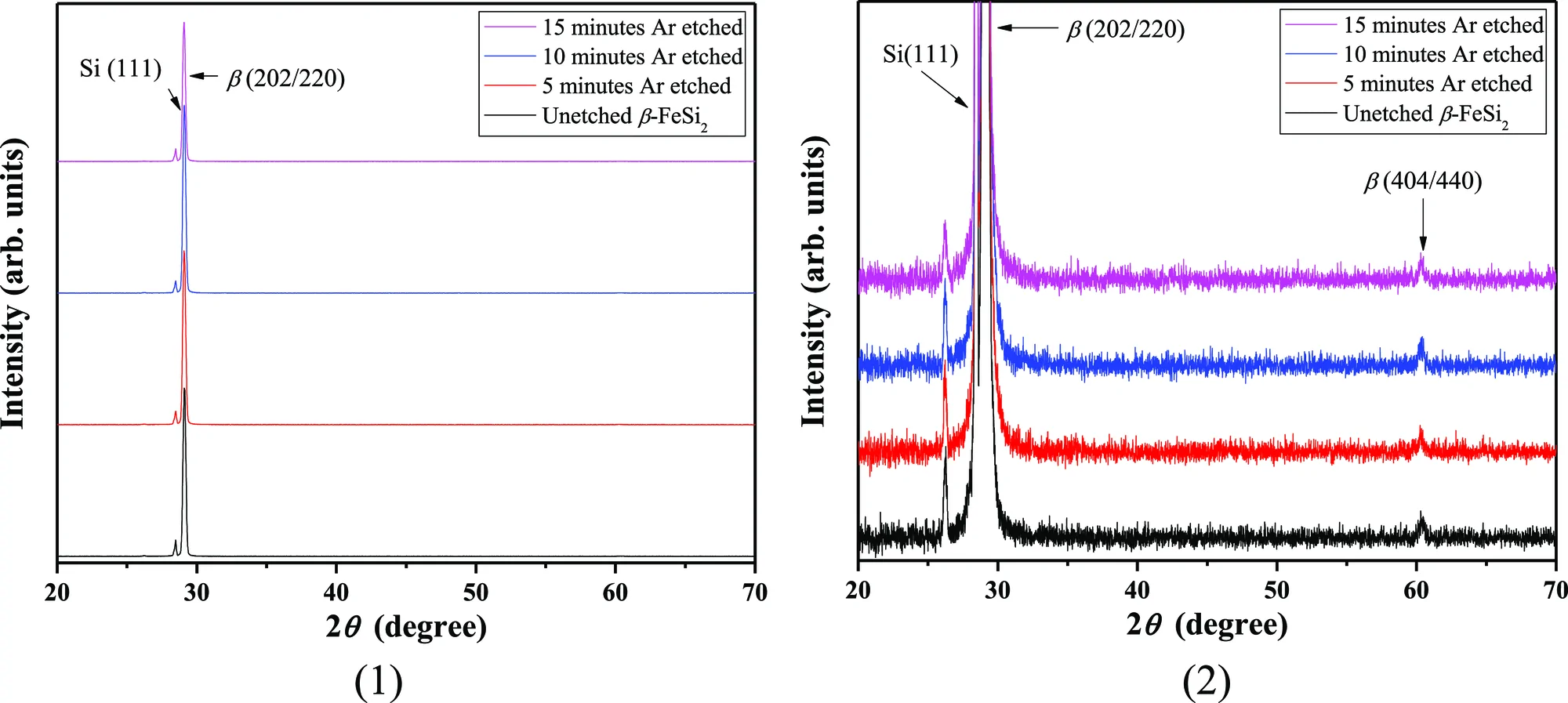
XRD patterns in the unetched β-FeSi_2_ thin films and etched films after etching for durations of 5, 10, and 15 min: (1) full peak intensity and (2) a zoom-up view.

**Table 7:** Parameters from the Rietveld Analysis of XRD Patterns of Each *β*-FeSi_2_ Film

	as-created			5 min			10 min			15 min		
*hkl*	FWHM	2θ (°)	d*_hkl_*	FWHM	2θ (°)	d*_hkl_*	FWHM	2θ (°)	d*_hkl_*	FWHM	2θ (°)	d*_hkl_*
β(021)	0.3169	26.2124	3.3968	0.2456	26.2119	3.3969	0.1961	26.2150	3.3965	0.2372	26.1921	3.3994
Si(111)	0.1541	28.4408	3.1355	0.1597	28.4462	3.1350	0.1543	28.4419	3.1354	0.1386	28.4525	3.1343
β(202)	0.2169	29.0913	3.0669	0.2196	29.0765	3.0684	0.2182	29.0816	3.0679	0.2166	29.0648	3.0696
β(404)	0.2900	60.4921	1.5291	0.1604	60.2667	1.5343	0.1849	60.3058	1.5334	0.2258	60.2725	1.5342
lattice	a	b	c	a	b	c	a	b	c	a	b	c
	10.4974	7.5583	7.7500	10.5617	7.5403	7.8340	10.5592	7.5392	7.8129	10.5551	7.5471	7.8097

**Table 8:** Fitted Parameters from the Williamson–Hall Analysis

condition	strain (%)	crystallite size (nm)	weighted profile *R*-factor	bragg *R*-factor	goodness of fit
as-created	–0.0003	30.1701	9.669	3.900	1.30
5 min etching	–0.0015	27.3239	9.060	3.997	1.25
10 min etching	–0.0007	35.3211	9.101	3.808	1.29
15 min etching	–0.0004	33.6782	10.240	4.180	1.26

Using Williamson–Hall analysis on parameters acquired from the Rietveld refinement of the acquired spectra of β-FeSi_2_, the results are discussed.

### Analytical Discussion on Surface Morphology

The unetched β-FeSi_2_ thin films were examined in comparison to those after etching with Ar gas plasma generated by a microwave under the same power condition of 300 W and different etching durations of 5, 10, and 15 min. The surface area and side-view cross-section of the thin films were then analyzed. According to the surface area images of the β-FeSi_2_ thin films before and after etching, the morphology changed from the original cluster with a clear grain boundary to almost amorphous. In addition, more holes appeared on the surface, and the hole size increased with etching duration. This is likely due to the plasma process in which Ar gas plasma particles or atoms are accelerated and collide with the particles on the surface of the samples, causing the bonds on the film surface to break and atoms to volatilize. The volatilized atoms detached, resulting in surface changes, as shown in Figure 8.^53^ The increased etching of the surface originates from the increasing plasma saturation attributed to the OES, resulting in a higher plasma temperature and broader energy distribution.^54^ A longer etching duration also increases the likelihood of affecting the film area.^55^ Hence, the morphology of the films becomes less defined with a longer duration despite the boundary becoming sharper 5 min after the etching of the Ar plasma.

**Figure 8:**
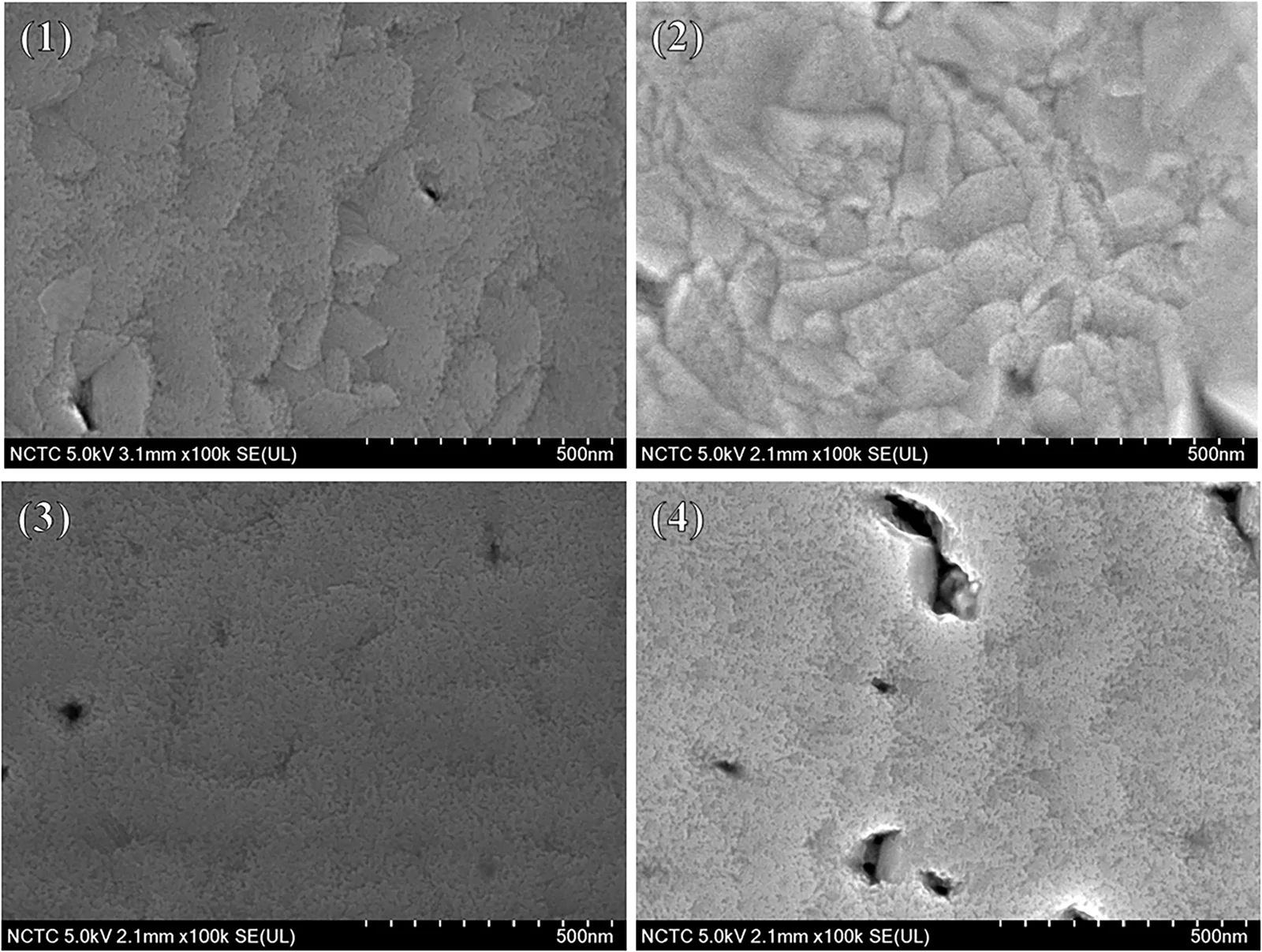
Surface-viewing FE-SEM micrographs of the surfaces of β-FeSi_2_ thin films under various conditions: (1) as-coated β-FeSi_2_ films, (2) after 5 min of etching, (3) after 10 min of etching, and (4) after 15 min of etching.

When analyzing the cross-section of a sample unetched by plasma, the average thickness of the film was roughly 563 nm. Then, plasma etching using a power of 300 W for 5 min caused the β-FeSi_2_ films to become relatively thinner, with an average film thickness of 553 nm. At surface etching durations of 10 and 15 min, the average thickness of the β-FeSi_2_ layers decreased to 537 and 508 nm, respectively. The decrease in the average film thickness, resulting from an increase in etching duration, is due to the interaction between the Ar plasma ions under the influence of the sheath potential and the surface atoms of the film, causing atomic detachment on the surface of the film.^20^ Therefore, as expected, a longer etching duration reduced the cross-sectional thickness of the etched β-FeSi_2_ layers, as shown in Figure 9.^54^ However, the observed thickness of the β-FeSi_2_ layers is less affected by plasma etching than that of our previously reported NC-FeSi_2_ films, despite being etched in the plasma for a longer duration, thus proving a substantially stronger bond between the film atoms.^20^

**Figure 9:**
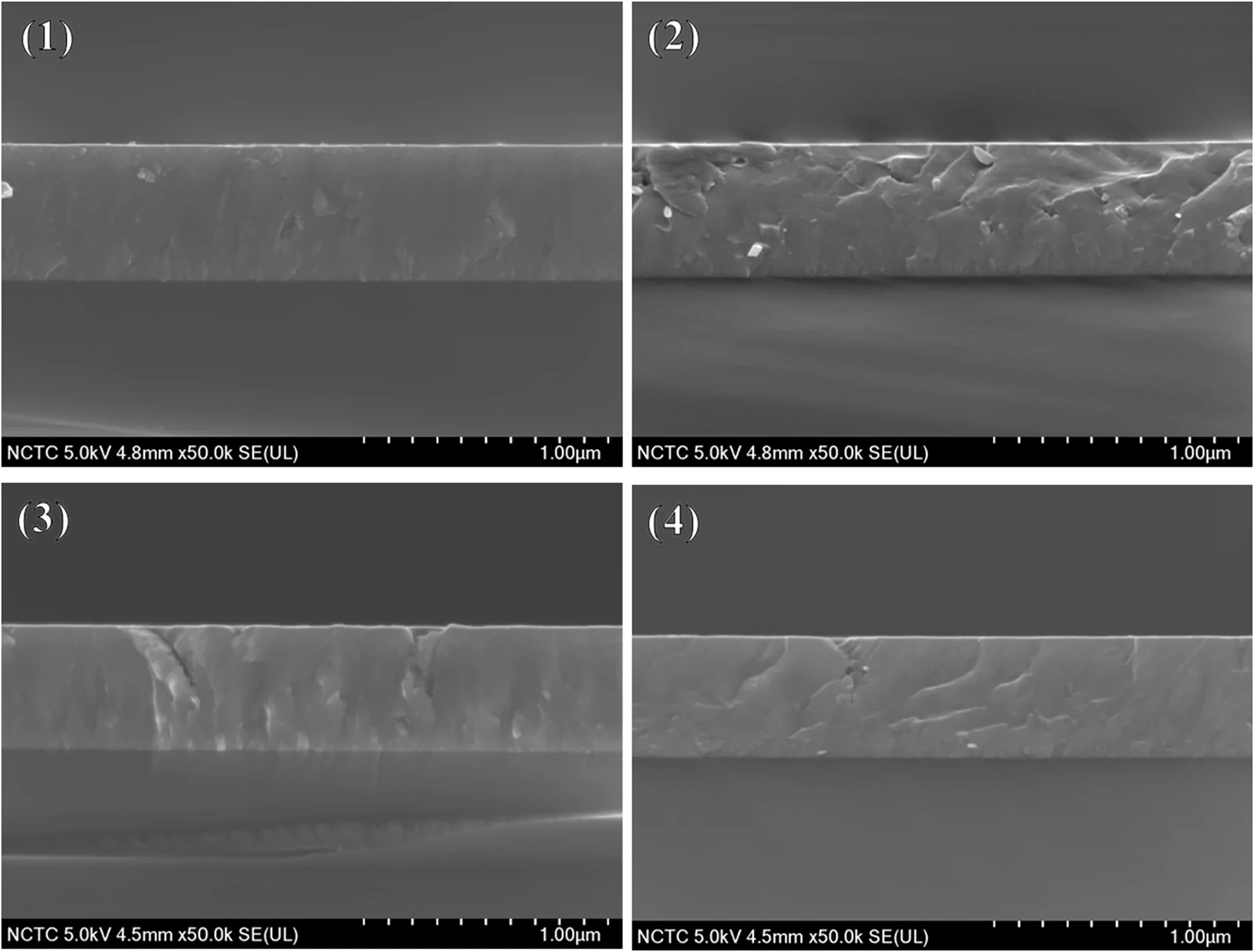
Cross-sectional FE-SEM photographs of the β-FeSi_2_ layers on Si substrate under various conditions: (1) as-coated β-FeSi_2_ films, (2) after 5 min of etching, (3) after 10 min of etching, and (4) after 15 min of etching.

The AFM analysis results for the β-FeSi_2_ thin films before and after microwave plasma etching with Ar gas plasma generated from a microwave at the same power and different etching durations were used to observe and compare the *R*_RMS_ values of the films. These values, expressed as *R*_RMS_, indicate the average deviation from the normal mean, providing an accurate roughness value. The *R*_RMS_ value derived from the surface topographies of the β-FeSi_2_ thin films before etching was 2.52 nm. When comparing the β-FeSi_2_ thin films after etching with Ar gas plasma generated from a microwave at etching durations of 5, 10, and 15 min, the average roughness values were 3.70, 3.74, and 4.10 nm, respectively. The trend indicates that the surfaces of the films were slightly roughened with an increase in the etching duration. This initially conflicted with the FE-SEM results but was expected due to the large holes on the surface of the etched films. The interaction resulted from the volatilization of surface atoms, causing the holes on the surface to enlarge with longer exposure to plasma, as shown in Figure 10.^53,54^ The increase in *R*_rms_, in contrast to the morphology from the surface FE-SEM, should be mainly due to pinholes that appear on the surface, which should influence the roughness detected by AFM, resulting in a smoother grain boundary. These holes swayed to the *R*_RMS_ values due to their drastic deviation, causing the overall value to increase.^20^ The mean roughness (*R*_a_) also increased from the initial value of the unetched films with longer etching durations. The main reason is the consistency of the contrast between the highest point (*R*_*z*_) and the lowest point (*R*_v_) throughout the surface. The skewness (*S*_sk_) and kurtosis (*S*_ku_) in Table 9 indicate that unetched films have a deep but ununiformly wide valley that is interrupted by unusual spikes.^56^ Afterward, the grain became partially less spiky, and the deep valleys were scattered more evenly as *S*_sk_ and *S*_ku_ declined, as the duration increased to 10 min. At 15 min etching, the parameters imply that the deep valley becomes much deeper and the grain becomes spikier due to the higher contrast between *R*_*z*_ and *R*_v_, as well as the increase in *S*_ku_, while the valley still becomes more uniform, as indicated by further decrease in *S*_sk_. The line profiles based on the topographies of the films, ranging from as-coated to after being etched for 15 min, are displayed in Figure 11.

**Figure 10:**
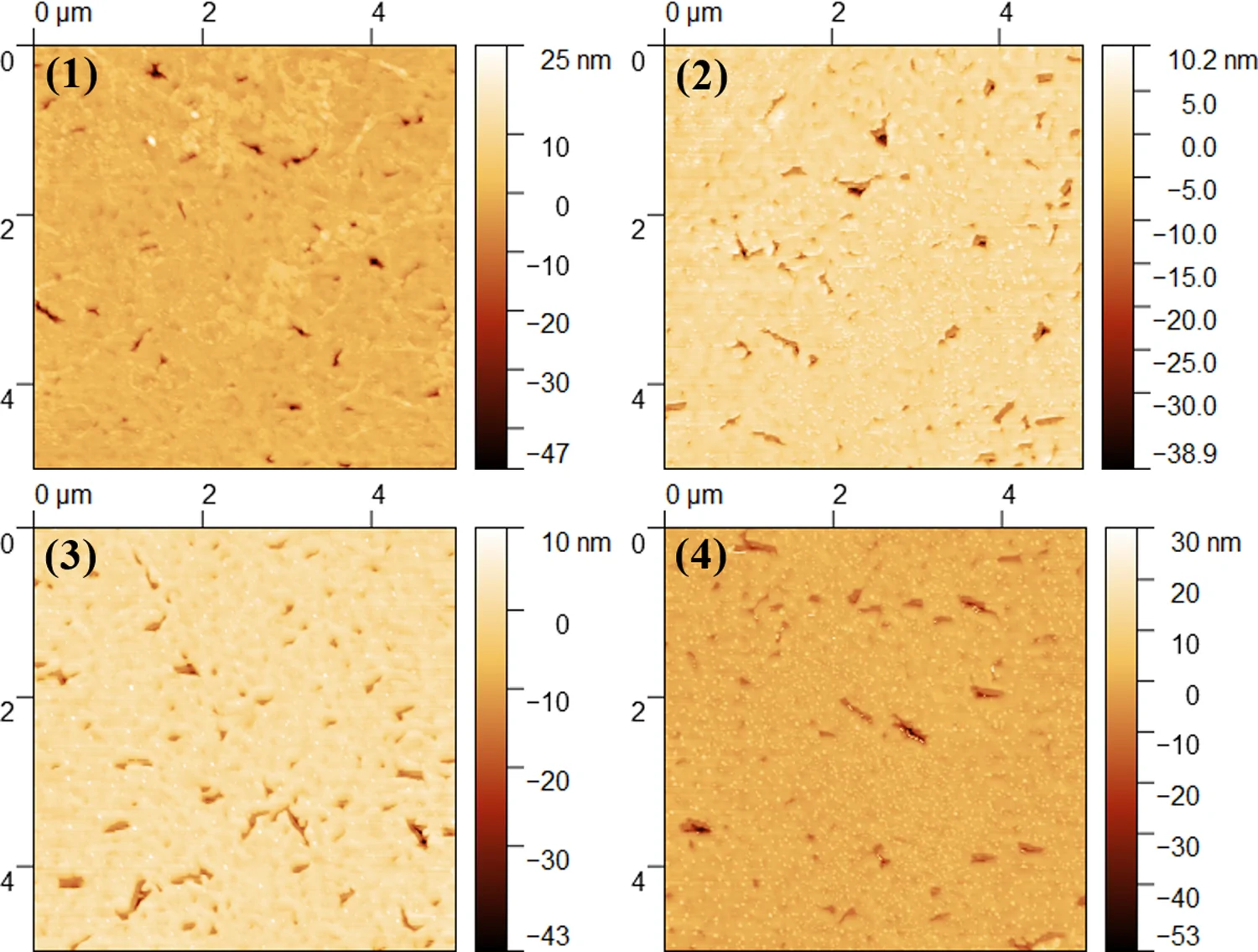
AFM topographies of the β-FeSi_2_ films: (1) as-coated β-FeSi_2_ films, (2) after 5 min of etching, (3) after 10 min of etching, and (4) after 15 min of etching.

**Figure 11:**
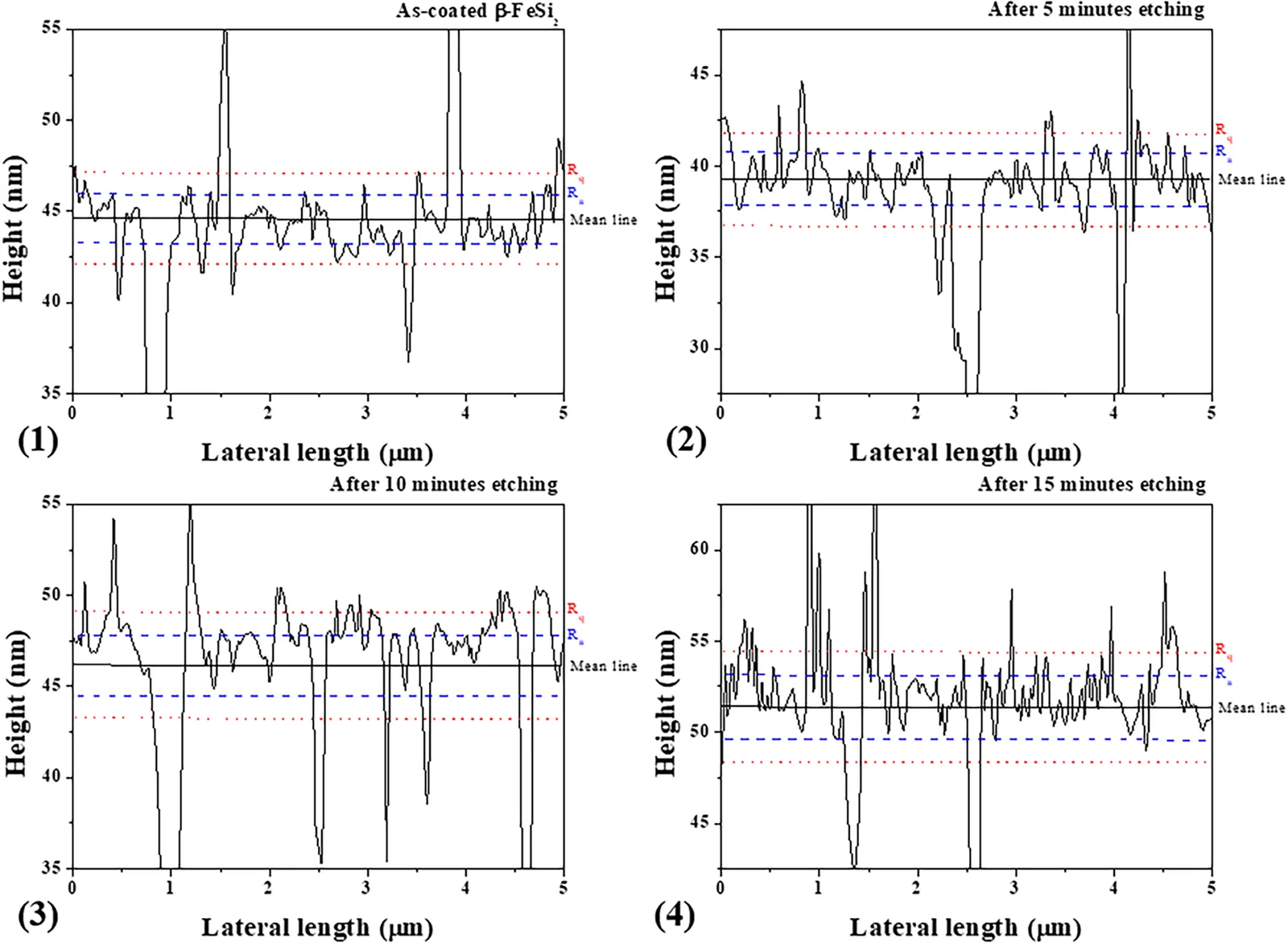
Line profiles of the β-FeSi_2_ films: (1) as-coated β-FeSi_2_ films, (2) after 5 min of etching, (3) after 10 min of etching, and (4) after 15 min of etching.

**Table 9:** Statistical Data Derived from AFM Topography of Each Film

condition	*R*_a_ (nm)	*R*_rms_ (nm)	*R*_*z*_ (nm)	*R*_v_ (nm)	*S*_sk_	*S*_ku_
unetched	1.43	2.52	68.36	44.65	–4.17	49.38
5 min	1.48	3.70	53.35	39.30	–3.04	22.69
10 min	1.59	3.74	58.93	46.21	–3.03	16.85
15 min	1.77	4.10	96.78	51.42	–1.80	25.70

### Analytical Discussion of Wettability

The results of the optical contact angle measurement on the β-FeSi_2_ thin films before and after microwave plasma etching with Ar gas plasma generated from a microwave at the same power and different etching times showed that the sessile contact angle of a DI water droplet on top of the β-FeSi_2_ thin film surface prior to surface etching was 96.13 ± 0.57°, indicating hydrophobicity. The DI water contact angles on the β-FeSi_2_ surfaces etched at 300 W for 5, 10, and 15 min were measured. The contact angles were found to slightly decrease with longer etching duration, remaining at around 73.98 ± 0.28°, 71.03 ± 0.49°, and 69.08 ± 0.58°, indicating hydrophilicity. As can be shown in Figure 12(2), the wetting properties of the surface changed from hydrophobicity to hydrophilicity after etching of the β-FeSi_2_ thin film. The hydrophilicity properties remained the same, as those shown in Figure 12(3,4). The wetting states of the material can be explained physically by the model proposed by Cassie–Baxter, which uses the simple principle of air bubbles in gaps on the rough surface to support the water droplets, in the explanation that could support the shift in the wetting state.^57,58^ Here, plasma etching results in the destruction of the air-gap structure via surface changes, causing the surface to lose its hydrophobicity, and the contact angle decreases with a longer etching time owing to the increased roughness.^59^ The alteration of chemical composition in the XPS section also contributes to the change in the hydrophobic surface into a hydrophilic one.^20^ Since the shift in the chemical composition is not as drastic as that in our previously reported NC-FeSi_2_ study, and the surface still possesses an air gap despite being less optimal, it leads to a subpar change in the contact angle with an increasingly longer duration.^20^ Although the shift in chemical composition is not as drastic as that in the previously reported NC-FeSi_2_ study, this range of contact angles is still typical for wetting materials.^20^ Based on the O 1s component peaks, the metal hydroxyl peak becomes significantly higher after longer etching compared to the preetched peak. Therefore, there is a high chance that new oxides or hydroxyl groups are formed. However, the wetting is slightly reduced even as the oxide peak becomes slightly lower at 15 min of etching, implying that either the reduction of hydrocarbon plays a bigger role in the enhancement of hydrophilicity or is due to more vacancies. In this regard of low chemical impact, the physical change from the wetting model could factor in the change in wettability, as the contact angle shows a significant negative correlation based on Pearson’s *r* of −0.9889, as shown in Figure 13. Physically, the initial hydrophobicity of the β-FeSi_2_ thin film can be explained using the air-gap model to support the water droplet, which disappears after the grain becomes less consistent, as indicated by the AFM results. This leads to the surface model becoming the Wenzel model, where water is in direct contact with the rough surface.^48^ Since the surface of the β-FeSi_2_ thin film turned hydrophilic, the rougher surface led to higher hydrophilicity, which may factor in the further increase in hydrophilicity at 15 min despite the smaller hydroxyl peak area.^48^ Based on the current results, the etched β-FeSi_2_ films can be considered as active surfaces for further bonding with polymers to form metallic-polymer layers for automotive, aerospace, and electronics applications due to their constant 70° hydrophilicity.^60^ Compared to conventional wetting coating materials, FeSi_2_ shares the usual advantages of being toxic-free and abundant in nature with TiO_2_ and SiO_2_. The nearly neutral wetting states of the Fe–Si composite also provide the potential to modify it to be either hydrophilic or hydrophobic, depending on the technique used for the modification.^61,62^

**Figure 12:**
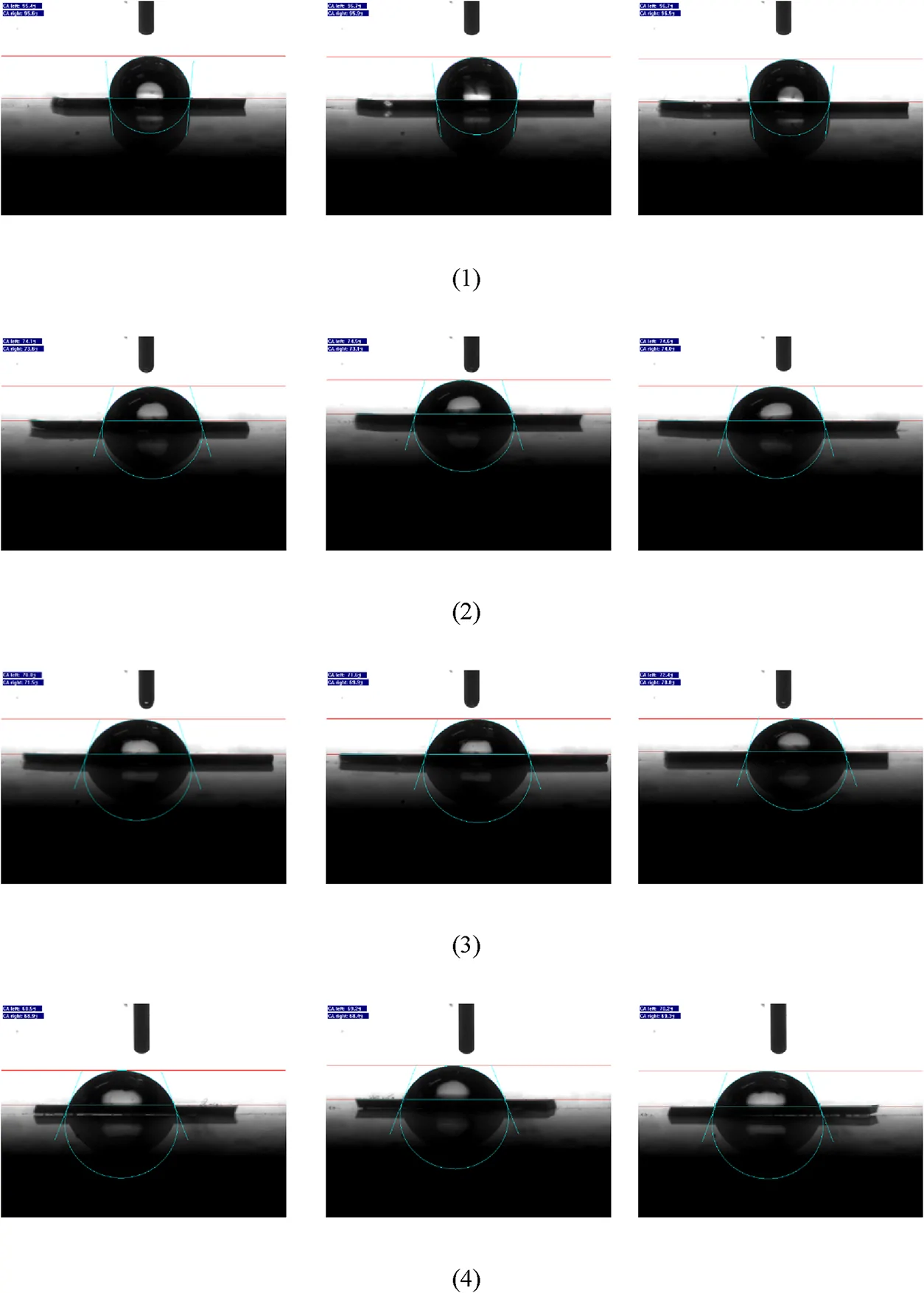
Images of the surface contact between DI water and β-FeSi_2_ films in the case of (1) as-coated β-FeSi_2_ films, (2) after 5 min of etching, (3) after 10 min of etching, and (4) after 15 min of etching.

**Figure 13:**
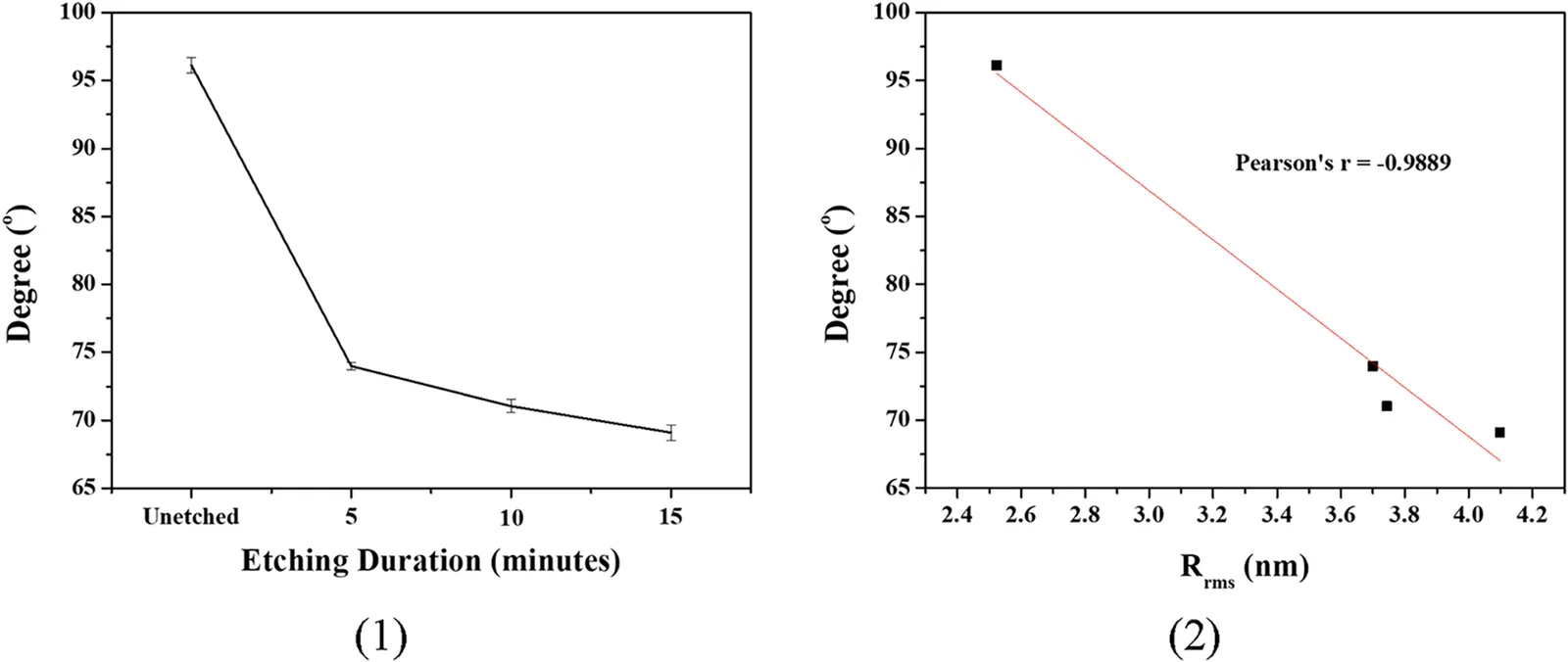
Contact angle values against (1) plasma etching duration and (2) *R*_rms_ values on β-FeSi_2_ films.

### Solid Mechanics Analytical Discussion

Figure 14 presents the load–depth curve for each β-FeSi_2_ film. The surface of the unetched β-FeSi_2_ thin film exhibited *H*_nano_ of around 14.058 GPa, with an *E*_r_ of around 204.670 GPa. A summary of the *H*_nano_ and *E*_r_ values for the β-FeSi_2_ films is presented in Table 10. The results show that the β-FeSi_2_ samples possess noticeably higher *H*_nano_ and *E*_r_ than the Fe_3_Si and NC-FeSi_2_ samples from our previous work and are closer to those of commercial coating materials, such as diamond-like carbon.^20,21,63,64^ In addition to the hardness nearly that of DLC, FeSi_2_ also possesses a high modulus, which is tougher than that of the other common self-cleaning materials. Additionally, FeSi_2_ should present better substrate adhesion when coated on an alloy or steel substrate due to its Fe composite nature. The etched β-FeSi_2_ films after 5 min displayed *H*_nano_ values of 15.649 and 220.110 GPa for the *E*_r_ value. With a longer etching duration of 10 min, the *H*_nano_ and *E*_r_ of the β-FeSi_2_ films were 15.851 and 216.027 GPa, respectively, which changed to 15.891 and 219.062 GPa after 15 min of plasma etching. The *H*_nano_ and *E*_r_ were almost the same, albeit slightly inclined, with a lower estimated standard deviation after Ar plasma etching. These results align with the inverse Hall–Petch principle, which shows an increasing trend when the surface defects, such as the oxide layer and unusual grain boundary slide after the surface change, as well as the shape factor of the grain, decrease (as discussed in the Analytical Discussion on Surface Morphology section and Figure 15).^65,66^ Following ion bombardment by Ar plasma, which increased saturation over time, the surface was damaged, resulting in less consistency in the surface structure and an increase in the standard deviation after increasing the etching duration.^66,67^ Moreover, the toughness (*H*/*E*) and plastic deformation resistance (*H* ^3^/*E* ^2^), as shown in Table 10, also show the same consistency, which further demonstrates that plasma deterioration is not significant. This implies that the contact angle of the β-FeSi_2_ films can be modified over time without compromising their mechanical properties.

**Figure 14:**
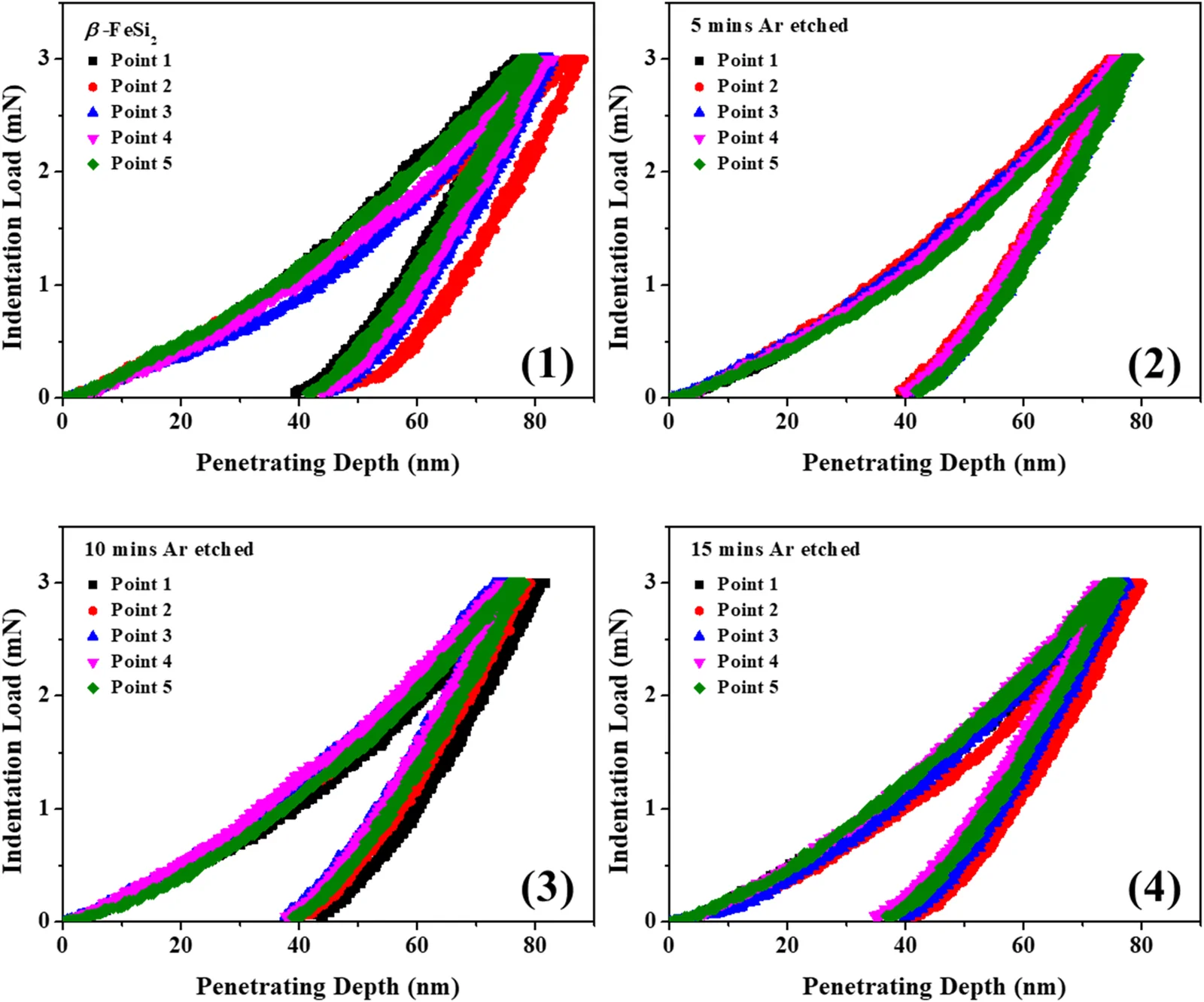
Load–depth plots for each β-FeSi_2_ film in the case of (1) as-coated β-FeSi_2_ films, (2) after 5 min of etching, (3) after 10 min of etching, and (4) after 15 min of etching.

**Figure 15:**
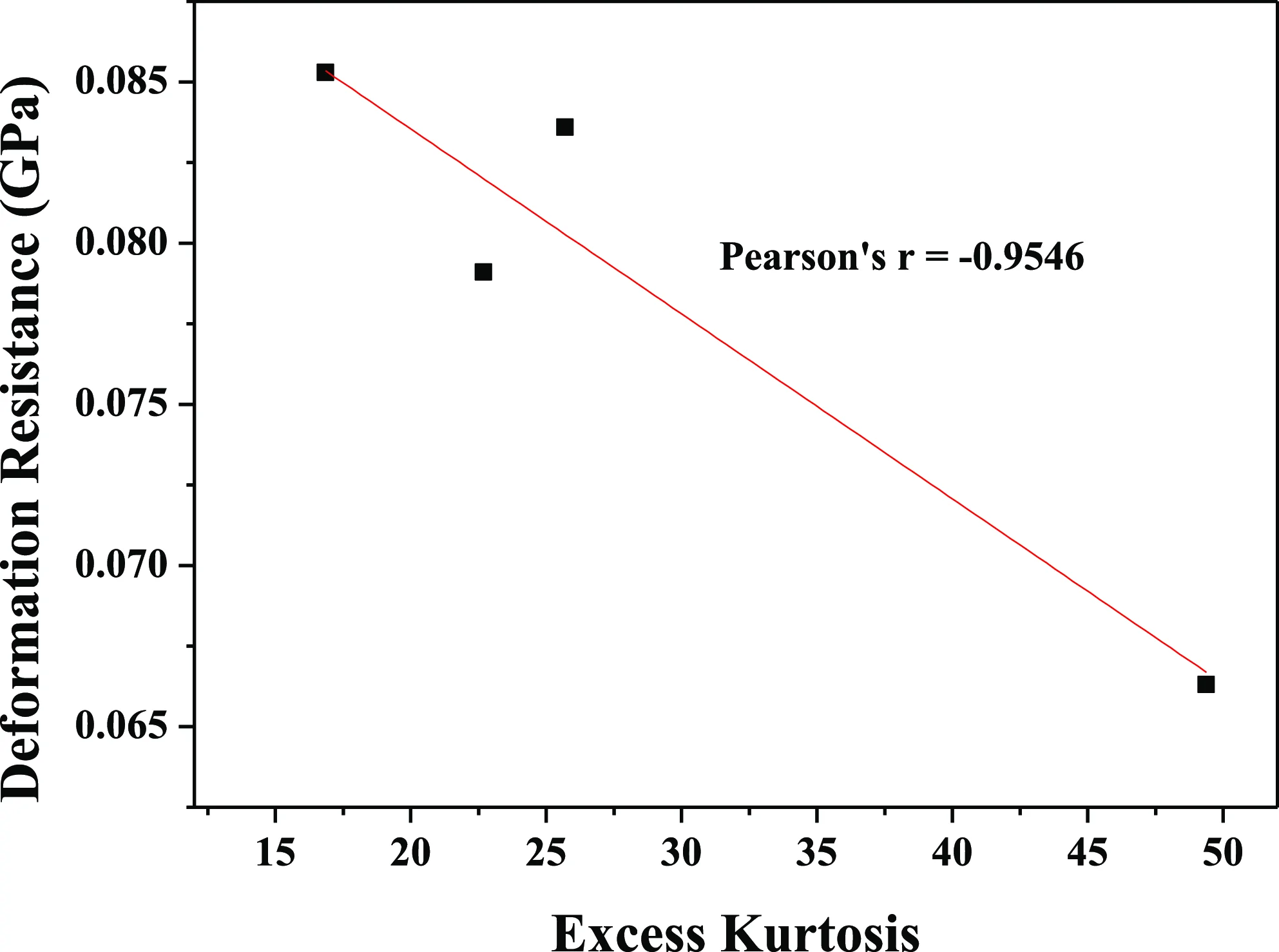
Deformation resistance against excess kurtosis values of the β-FeSi_2_ films.

**Table 10:** Values Representing the Solid Mechanics of the *β*-FeSi_2_ Films under Various Conditions Derived from the Load–Depth Curves

sample	*H*_nano_ (GPa)	*E*_r_ (GPa)	*H*_nano_/*E*_r_	*H*_nano_^3^/*E*_r_^2^ (GPa)
unetched β-FeSi_2_	14.058 ± 1.292	204.670 ± 9.520	0.0687	0.0663
Ar-etched (300 W; 5 min)	15.649 ± 0.450	220.110 ± 3.798	0.0711	0.0791
Ar-etched (300 W; 10 min)	15.851 ± 1.050	216.027 ± 4.503	0.0734	0.0853
Ar-etched (300 W; 15 min)	15.891 ± 1.136	219.062 ± 3.300	0.0725	0.0836

## Conclusion

In this study, the effects of microwave-generated Ar gas plasma etching on β-FeSi_2_ thin films are examined by subjecting them to Ar plasma etching for different durations of 5, 10, and 15 min. The key findings include plasma diagnosis, chemical composition, structural analysis, surface morphology, wettability, and mechanical properties. The OES spectra of Ar plasma used for etching with 300 W power over 5–15 min showed excited Ar and oxygen atoms, where *T*_e_ decreased from 0.287 to 0.283 eV with increased etching time, while *n*_e_ increased. These plasma parameters are consistent with the cold plasma characteristics. XPS analysis revealed alterations in the surface atomic concentration of β-FeSi_2_ thin films, with decreased C 1s and increased O 1s concentrations over a longer duration, implying the removal of surface contaminants and accumulation of hydrophilic oxide and hydroxyl by increasing the plasma etching duration until 15 min, where the O 1s decreased. Meanwhile, the XRD patterns showed no significant changes in the β-FeSi_2_ films etched for any duration. The FE-SEM and AFM analyses revealed a transition from defined grain boundaries to a less defined morphology with increasing *R*_RMS_ (from 2.52 nm as-created to 4.10 nm after 15 min of etching) due to the formation of larger holes and a reduction in the film thickness (from 563 nm as-created to 508 nm after 15 min of etching). Plasma etching reduced the contact angle from 96.13 ± 0.57° (hydrophobic) to 69.08 ± 0.58° (hydrophilic) after 15 min of etching due to a combination of the change in morphology, leading to a collapse in the gap that supported the droplet, and chemical composition changes in the surface. The values of *H*_nano_ and *E*_r_ remained relatively consistent, with slight deviations attributed to the surface contamination layer and defect removal during the initial etching, where *H*_nano_ increased from 14.058 to 15.891 GPa, while *E*_r_ increased from 204.670 to 219.062 GPa. These results highlight the impact of plasma etching duration on the oxidation, morphology, composition, and wettability of the β-FeSi_2_ films while preserving their mechanical integrity, allowing further development into coating materials for use in metal–polymer materials.
